# Improved Generation of Human Induced Pluripotent Stem Cell-Derived Cardiac Pacemaker Cells Using Novel Differentiation Protocols

**DOI:** 10.3390/ijms23137318

**Published:** 2022-06-30

**Authors:** Fabrice F. Darche, Nina D. Ullrich, Ziqiang Huang, Michael Koenen, Rasmus Rivinius, Norbert Frey, Patrick A. Schweizer

**Affiliations:** 1 Department of Cardiology, Angiology and Pneumology, Heidelberg University Hospital, 69120 Heidelberg, Germany; koenen@mpimf-heidelberg.mpg.de (M.K.); rasmus.rivinius@med.uni-heidelberg.de (R.R.); norbert.frey@med.uni-heidelberg.de (N.F.); patrick.schweizer@med.uni-heidelberg.de (P.A.S.); 2 German Center for Cardiovascular Research (DZHK), Partner Site Heidelberg/Mannheim, 69120 Heidelberg, Germany; nina.ullrich@physiologie.uni-heidelberg.de; 3 Institute of Physiology and Pathophysiology, Heidelberg University, 69120 Heidelberg, Germany; 4 EMBL Imaging Centre, European Molecular Biology Laboratory (EMBL), Meyerhofstraße 1, 69117 Heidelberg, Germany; ziqiang.huang@embl.de; 5 Department of Molecular Neurobiology, Max-Planck-Institute for Medical Research, Jahnstraße 29, 69120 Heidelberg, Germany

**Keywords:** hiPSC, cardiac pacemaker cells, novel differentiation protocols

## Abstract

Current protocols for the differentiation of human-induced pluripotent stem cells (hiPSC) into cardiomyocytes only generate a small amount of cardiac pacemaker cells. In previous work, we reported the generation of high amounts of cardiac pacemaker cells by co-culturing hiPSC with mouse visceral endoderm-like (END2) cells. However, potential medical applications of cardiac pacemaker cells generated according to this protocol, comprise an incalculable xenogeneic risk. We thus aimed to establish novel protocols maintaining the differentiation efficiency of the END2 cell-based protocol, yet eliminating the use of END2 cells. Three protocols were based on the activation and inhibition of the Wingless/Integrated (Wnt) signaling pathway, supplemented either with retinoic acid and the Wnt activator CHIR99021 (protocol B) or with the NODAL inhibitor SB431542 (protocol C) or with a combination of all three components (protocol D). An additional fourth protocol (protocol E) was used, which was originally developed by the manufacturer STEMCELL Technologies for the differentiation of hiPSC or hESC into atrial cardiomyocytes. All protocols (B, C, D, E) were compared to the END2 cell-based protocol A, serving as reference, in terms of their ability to differentiate hiPSC into cardiac pacemaker cells. Our analysis revealed that protocol E induced upregulation of 12 out of 15 cardiac pacemaker-specific genes. For comparison, reference protocol A upregulated 11, while protocols B, C and D upregulated 9, 10 and 8 cardiac pacemaker-specific genes, respectively. Cells differentiated according to protocol E displayed intense fluorescence signals of cardiac pacemaker-specific markers and showed excellent rate responsiveness to adrenergic and cholinergic stimulation. In conclusion, we characterized four novel and END2 cell-independent protocols for the differentiation of hiPSC into cardiac pacemaker cells, of which protocol E was the most efficient.

## 1. Introduction

Diseases that impair the proper function of the human sinus node (hSAN), are currently treated by cardiac pacemaker devices. However, they bear a lot of disadvantages like lead dysfunction, the need for frequent battery change, perioperative infections requiring total system explantation and the lack of response to the autonomic nervous system [1,2]. Therefore, the implementation of new therapeutic strategies, which bypass the side effects of cardiac pacemaker devices, is paramount. In this context, the development of a biological pacemaker is very promising [3] and can be achieved by approaches consisting of cell [4,5] and genetic therapeutic strategies [5]. Due to the fact that genetic strategies bear the risk of oncogenicity [6,7], cell strategies may be the most auspicious approach. The discovery of human-induced pluripotent stem cells (hiPSC) [8] provided access to cell therapeutic strategies, especially in the field of regenerative medicine [9]. Based on their pluripotency, hiPSC can be differentiated into all types of somatic cells [8]. Protocols for the differentiation of hiPSC or human embryonic stem cells (hESC) into cardiomyocytes have already been established [10,11,12,13,14,15,16]. 

However, although particularly efficient regarding the differentiation of hiPSC or hESC into cardiomyocytes of ventricular phenotype, the amount of cardiac pacemaker cells received remains, at approximately 10%, very low [17]. In this regard, we established a pacemaker-specific protocol that co-cultures hiPSC with mouse visceral endoderm-like cells (END2 cells) followed by growth in fetal bovine serum (FBS)-enriched culture medium to promote differentiation toward cardiac pacemaker cells [4]. Importantly, we increased the actual yield of cardiac pacemaker cells to 63.4% without the need for recurring genetic manipulations [4]. However, the use of mouse END2 cells bears a xenogeneic risk, which might raise safety issues regarding future applications in humans [18] as well as the restricted scalability of an END2 cell-based co-culture system [19]. Recently, it was shown that inhibition of NODAL by the small molecule SB431542 during deactivation of the Wingless/Integrated (Wnt) signaling pathway by IWR-1 increases the fraction of hiPSC-derived cardiac pacemaker-like cells [20]. Similar results were obtained by simultaneous application of SB431542 and the Wnt inhibitor IWP-2 [21]. Moreover, a second application of the Wnt activator CHIR99021 as well as administration of retinoic acid (RA) after Wnt inhibition by IWR-1 promoted differentiation of hiPSC toward a more atrial- and epicardial-like fate with a reduced proportion of ventricular-like cardiomyocytes and correlated with high expression levels of T-box transcription factor 18 (*Tbx18*) [22], known to induce the development of cardiac pacemaker tissue [23,24]. Furthermore, the manufacturer STEMCELL Technologies recently established a protocol for the differentiation of hiPSC into cardiomyocytes with predominantly atrial-like characteristics. 

Due to the limitations in future medical applications associated with the use of END2 cells [18,19], we aimed to develop new protocols for the differentiation of hiPSC into cardiac pacemaker cells that substitute END2 cell-based co-culture. In these new differentiation protocols, we used culture media and components that induce activation or inhibition of Wnt signaling [15,16,17], supplemented either with the NODAL inhibitor SB431542 or with RA and a second application of the Wnt activator CHIR99021 or combining all three components to enhance hiPSC differentiation toward cardiac pacemaker cells. The following differentiation step in FBS-enriched medium, however, remained unchanged [4]. Moreover, the protocol from the manufacturer STEMCELL Technologies was validated for its ability to differentiate hiPSC into cardiac pacemaker cells. Finally, we compared these novel protocols in terms of differentiation efficiency with the END2 cell-based pacemaker-specific protocol [4].

## 2. Results

### 2.1. Novel Cell Culture Protocols for the Differentiation of hiPSC into Cardiac Pacemaker Cells

**Figure 1:**
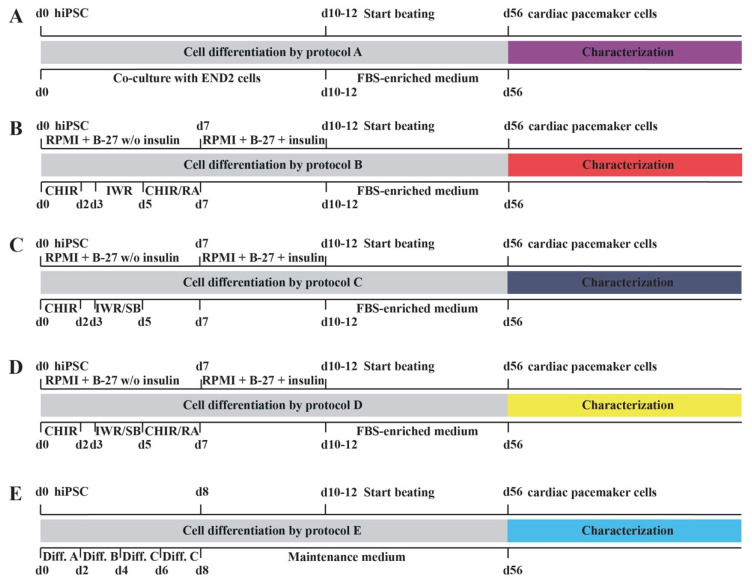
Schematic overview of the novel differentiation protocols. (**A**): Reference protocol A based on a co-culture of hiPSC and END2 cells. (**B**–**D**): Novel protocols (**B**–**D**) based on activation (6 µM CHIR 99021 on days 0 + 1) and inhibition (5 µM IWR-1 on days 3 + 4) of Wingless/Integrated (Wnt) signaling with following modifications for each protocol: (**B**): Additional administration of 1 µM RA and 5 µM CHIR 99021 on days 5 + 6. (**C**): Additional administration of 5 µM of the NODAL inhibitor SB431542 on days 3 + 4. (**D**): Additional administration of 5 µM of the NODAL inhibitor SB431542 on days 3 + 4 as well as of 1 µM RA and 5 µM CHIR 99021 on days 5 + 6. (**E**): Protocol of the manufacturer STEMCELL Technologies consisting of three differentiation compounds (**A**–**C**), followed by culture in Maintenance medium. Abbreviations: hiPSC = human induced pluripotent stem cells, END2 cells = mouse visceral endoderm-like cells, FBS = fetal bovine serum, d = day, RPMI = RPMI-1640, B-27 = B-27 supplement, w/o = without, CHIR = CHIR 99021, IWR = IWR-1, RA = retinoic acid, SB = SB431542, Diff. A, B and C = differentiation compounds A, B and C.

Figure 1 gives a summary of the various protocols used for the differentiation of hiPSC into cardiac pacemaker cells. These novel protocols B–E were compared to the already published reference protocol A [4] regarding differentiation efficiency of hiPSC toward the cardiac pacemaker cell lineage. Cell differentiation by reference protocol A was initiated by a co-culture of hiPSC and END2 cells [4], whereas the novel protocols B–D were based on activation and inhibition of Wnt signaling pathways [15,16,17] supplemented either with RA and a second application of the Wnt activator CHIR99021 [22] (protocol B) or with the NODAL inhibitor SB431542 [20] (protocol C) or with all three substances (protocol D). Importantly, after initiation of spontaneous beating, cells were transferred to an FBS-enriched culture medium, identical for the protocols A–D. Differentiation protocol E consisted of the newly developed STEMdiff™ Atrial Cardiomyocyte Differentiation Kit from STEMCELL Technologies. Approximately 50% of initially seeded hiPSC were spontaneously beating if differentiated by protocols B–D. These results were similar to those achieved by reference protocol A [4]. In contrast, protocol E yielded at least 80% spontaneously beating areas.

### 2.2. Proliferation and Survival Analysis of Cardiac Pacemaker Cells 

**Figure 2:**
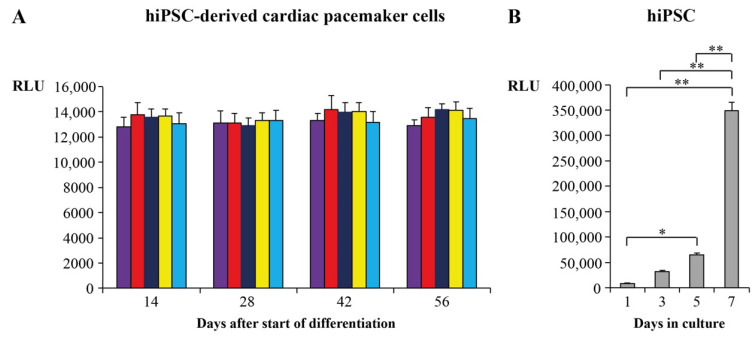
ATP-based cell proliferation assays. (**A**): hiPSC-derived cardiac pacemaker cells differentiated by protocols A (violet), B (red), C (dark blue), D (yellow) and E (blue). (**B**): hiPSC. Data are provided as means ± SEM. * *p* < 0.05, ** *p* < 0.01. Abbreviations: hiPSC = human induced pluripotent stem cells, RLU = relative light units, ATP = adenosine triphosphate.

We used ATP-based proliferation assays to evaluate whether hiPSC-derived cardiac pacemaker cells differentiated by protocols B–E continue to differentiate. Relative light units (RLU) of cardiac pacemaker cells differentiated by protocols B-E were therefore compared to RLU of hiPSC and cardiac pacemaker cells differentiated by protocol A, which were already determined in our previous work [4]. Results of the ATP-based assays are depicted in Figure 2. Compared to hiPSC, which showed an exponential proliferation (Figure 2B) [4], hiPSC-derived cardiac pacemaker cells showed constant RLU, hence demonstrating that they did not proliferate any more during and at the end of differentiation (Figure 2A). Of note, results were similar for all novel differentiation protocols (B–E) and comparable to those of reference protocol A [4]. Moreover, as the RLU remained constant during differentiation, we can also conclude that there was no relevant cell death, thus indicating that survival of hiPSC-derived cardiac pacemaker cells was not significantly impaired.

### 2.3. Valuation of the New Protocols’ Differentiation Efficiency toward Cardiac Pacemaker Cells by qRT-PCR 

**Figure 3:**
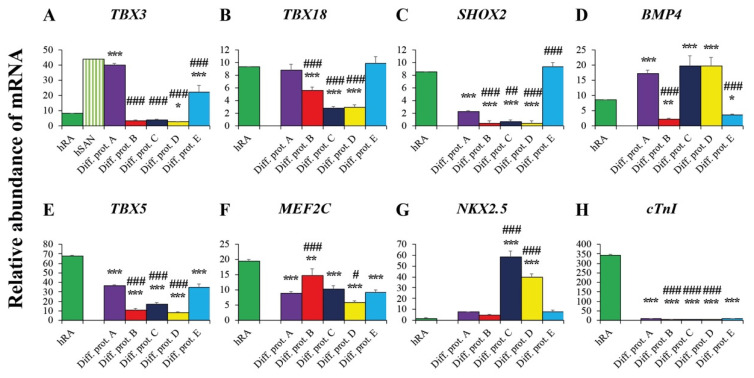
Gene expression of pacemaker-specific and myocardium-related transcription factors in cardiac pacemaker cells. Abundance of mRNA transcripts (in%) relative to gene expression of the housekeeping genes *GAPDH/HPRT1/ACTB*. (**A**–**D**): Pacemaker-specific transcription factors. (**E**–**G**): Myocardium-related transcription factors. (**H**): Myocardial marker troponin I (*cTnI*). Data are provided as means ± SEM. * *p* < 0.05, ** *p* < 0.01, *** *p* < 0.001, Diff. prot. A–E versus hRA. # *p* < 0.05, ## *p* < 0.01, ### *p* < 0.001, Diff. prot. B–E versus Diff. prot. A. Abbreviations: hRA: human right atrium, hSAN: human sinoatrial node, Diff. prot. = differentiation protocol.

**Figure 4:**
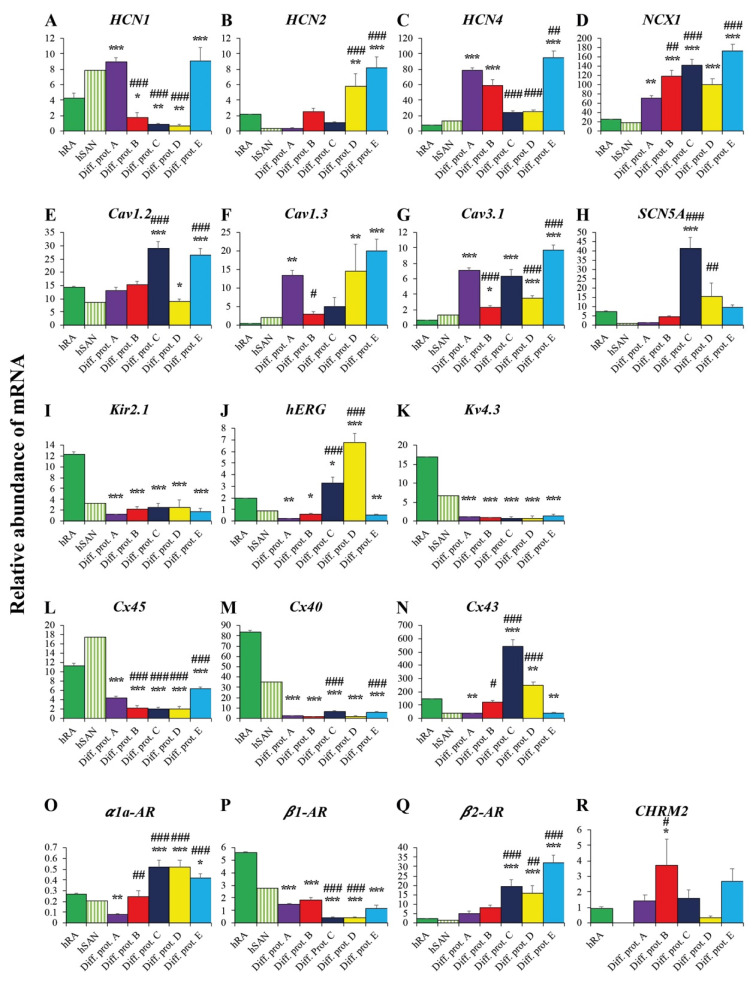
Gene expression of ion channels, transporters, connexins, adrenergic and cholinergic receptors in cardiac pacemaker cells. Abundance of mRNA transcripts (in%) relative to gene expression of the housekeeping genes *GAPDH/HPRT1/ACTB*. (**A**–**C**): *HCN* channels. (**D**): Na-Ca exchanger *NCX1*. (**E**–**G**): calcium channels. (**H**): sodium channel *SCN5A*. (**I**–**K**): potassium channels. (**L–N**): connexins. (**O**–**R**): adrenergic and cholinergic receptors. Data are provided as means ± SEM. * *p* < 0.05, ** *p* < 0.01, *** *p* < 0.001, Diff. prot. A–E versus hRA. # *p* < 0.05, ## *p* < 0.01, ### *p* < 0.001, Diff. prot. B–E versus Diff. prot. A. Abbreviations: hRA: human right atrium, hSAN: human sinoatrial node, Diff. prot. = differentiation protocol.

Our aim was to estimate the efficiency of the novel protocols to differentiate hiPSC into cardiac pacemaker cells. For that purpose, two distinct references were used to evaluate the gene expression profile of cardiac pacemaker cells generated by the novel protocols B-E. As the first reference, we used the transcription profile obtained from cardiac pacemaker cells generated by protocol A, based on co-culture with END2 cells [4]. Statistically significant differences regarding transcription levels between each of the novel protocols and reference protocol A are marked by a hashtag (#) (Figure 3 and Figure 4). As a second reference, we chose the transcription profile of the human right atrium (hRA) and human sinoatrial node (hSAN) [4]. Statistically significant differences between each of the differentiation protocols and hRA are indicated by an asterisk (*) (Figure 3 and Figure 4). Of note, hSAN transcript levels were not determined, but calculated by multiplying the hRA transcript levels with a hSAN/hRA ratio [4], on the basis of data published by Chandler et al. [25].

#### 2.3.1. Pacemaker-Specific Transcription Factors

The pacemaker-specific transcription factors T-box transcription factors 3 (*Tbx3*) and 18 (*Tbx18*) play an important role in the development of cardiac pacemaker tissue through activation of nodal-specific genetic pathways leading to SAN formation and suppression of pathways involved in the generation of working myocardium [23,26]. Cardiac pacemaker cells generated from hiPSC by protocol E showed *TBX18* transcript levels similar to those in hRA and cells differentiated by reference protocol A (Figure 3B). In contrast, *TBX3* transcript levels were significantly higher in cardiac pacemaker cells generated by protocol E than in hRA but lower than in cells differentiated according to reference protocol A (Figure 3A). Importantly, cardiac pacemaker cells differentiated by protocols B, C and D showed significantly lower *TBX3* and *TBX18* gene expression levels than hRA and cells generated by reference protocol A (Figure 3A,B). We also determined the transcript levels of the transcription factor SHOX2 and its target Bmp4, both involved in the development of cardiac pacemaker cells [27]. *SHOX2* was highly transcribed in cardiac pacemaker cells generated by protocol E with levels similar to those in hRA and significantly higher than those in cells differentiated by reference protocol A (Figure 3C). In contrast, cells generated by protocols B, C and D only displayed very low gene expression levels of *SHOX2* (Figure 3C). *BMP4*, however, was markedly transcribed in cardiac pacemaker cells generated by protocols C and D reaching levels similar to those in cells differentiated by reference protocol A and exceeding those in hRA (Figure 3D). In cells generated by protocols B and E, *BMP4* levels remained relatively low though (Figure 3D).

#### 2.3.2. Transcription Factors and Markers of the Working Myocardium

T-box transcription factor 5 (Tbx5), homeobox protein Nkx2.5 and myocyte enhancer factor 2C (Mef2c) promote differentiation into working myocardium [28]. While Tbx5 and Nkx2.5 are both involved in the development of ventricular cardiomyocytes [29], Nkx2.5 was shown to reduce SAN formation in embryonic mice [30], indicating a more suppressive role for Nkx2.5 on cardiac pacemaker-specific cell differentiation. 

In this regard, we observed significantly lower expression levels of *TBX5* and *MEF2C* in cardiac pacemaker cells generated by the new protocols than in hRA (Figure 3E,F). *NKX2.5* was scarcely transcribed in cardiac pacemaker cells generated by protocols B and E, with levels similar to those observed in hRA and in cells differentiated by protocol A (Figure 3G). In contrast, cardiac pacemaker cells generated by protocol C and D revealed high *NKX2.5* transcript levels (Figure 3G). The fact that the myocardial marker *cardiac Troponin I (cTNI)* was barely detectable in cardiac pacemaker cells generated by differentiation protocols B, C, D and E, impressively underlines the high selectivity of these protocols toward the generation of cardiac pacemaker cells (Figure 3H).

#### 2.3.3. Ion Channels and Transporters

The funny current (I_f_) is one of the major cardiac pacemaker currents [31] generated by hyperpolarization-activated cyclic nucleotide channels (HCN). It is importantly involved in SAN pacemaker activity [32,33] and early embryonic heart development [34,35,36]. SAN-specific HCN-isoforms are first of all HCN4 and to a lower extent HCN1 [25,36]. HCN2, by contrast, is predominantly expressed in atrial cells [25]. There is, however, some evidence that it also contributes to SAN function [37,38]. hiPSC-differentiated cardiac pacemaker cells by protocol E showed very high transcription levels of *HCN1* (Figure 4A) and *HCN4* (Figure 4C), clearly exceeding those of hRA and hSAN. *HCN4* was even higher transcribed in cardiac pacemaker cells generated by protocol E than in cells differentiated by reference protocol A (Figure 4C). Interestingly, cells generated by protocols B, C and D showed *HCN4* transcript levels exceeding those of hRA or hSAN, although they still ranged below those of cardiac pacemaker cells generated by protocols A and E (Figure 4C). This was in fact different from *HCN1*, where cells generated by protocols B, C and D showed lower transcript levels than hRA or hSAN (Figure 4A). *HCN2*, by contrast, was abundantly transcribed in cardiac pacemaker cells generated by protocols D and E, displaying significantly higher gene expression levels than hRA and cells derived from protocol A (Figure 4B). 

The sodium-calcium-exchanger 1 (NCX1), also crucial for cardiac pacemaking [39], displayed high transcription levels in all cardiac pacemaker cells, independent of the applied protocols and surpassed transcript levels assessed in hRA or hSAN (Figure 4D). However, the highest *NCX1* transcript levels were observed in cells generated by protocol E (Figure 4D). L-type (Ca_v_1.2 and Ca_v_1.3) and t-type (Ca_v_3.1) calcium channels as part of the membrane clock system contribute importantly to the pacemaker current [40,41]. Accordingly, cardiac pacemaker cells generated by the differentiation protocols A, B, C, D and E showed transcript levels of Ca_v_1.2, Ca_v_1.3 and Ca_v_3.1 similar or even higher than those observed in hRA or hSAN (Figure 4E–G). Interestingly, Ca_v_1.2 showed the highest transcript levels in cells generated by protocol C (Figure 4E), while cells generated by protocol E led to the highest levels of Ca_v_1.3 and Ca_v_3.1 transcripts (Figure 4F,G). 

Transcription of the major depolarizing cardiac sodium current Na_v_1.5, responsible for the action potential upstroke especially in ventricular-like cardiomyocytes [42], remained low, with the exception of cells generated by protocols C and D with levels comparable to those in hRA or hSAN (Figure 4H). Transcription of the inward rectifier K_ir_2.1 and potassium channel K_v_4.3 was low in all cardiac pacemaker cells regardless of the used differentiation protocol, showing transcript levels below those in hRA and comparable to those in the hSAN (Figure 4I,K). As distinct from cardiac pacemaker cells generated by protocols C and D, we observed only minor *hERG* transcript levels in cells differentiated by protocols A, B and E (Figure 4J). 

#### 2.3.4. Connexins

Propagation of electrical signals within cardiac tissue is ensured by connexins (Cx) [43]. Cx45 is associated with the SAN and the conduction system [43], while Cx40 and Cx43 belong to the working myocardium [44,45]. *Cx45* transcript levels were highest in cardiac pacemaker cells generated by protocol E, clearly exceeding levels in cells differentiated by reference protocol A (Figure 4L). In contrast, cells generated by protocols B, C and D showed significantly lower levels (Figure 4L). Independently of the used differentiation protocol, *Cx40* was transcribed on negligible levels in all cells far below hRA or hSAN levels (Figure 4M). Similarly, ventricle-specific *Cx43* was scarcely transcribed in cells generated by protocols A and E with levels similar to those in the hSAN (Figure 4N), while significant higher levels emerged in cells generated by protocols B, C and D (Figure 4N).

#### 2.3.5. Adrenergic and Cholinergic Receptors

Adrenergic and cholinergic receptors are required for beating rate modulation of cardiac pacemaker cells [46,47]. Except for cells generated by protocols A and B, cardiac pacemaker cells revealed transcription levels of the *α1a-adrenoreceptor (AR)* higher than in hRA or hSAN (Figure 4O). Similar results were obtained for the *β2-AR* (Figure 4Q). This was different from *β1-AR*, where the highest transcript levels assessed in cardiac pacemaker cells generated by protocols A, B and E, still ranged below hRA or hSAN levels (Figure 4P). Cholinergic receptor *CHMR2* transcription was highest in cells generated by protocols B and E and clearly surmounted hRA levels (Figure 4R). 

### 2.4. Comparison of the Differentiation Protocols Regarding the Transcription of Cardiac Pacemaker-Specific Genes

Table 1 represents an overview of the number of cardiac pacemaker-specific genes transcribed in cells generated by the differentiation protocols A-E. Genes were marked by a “+” sign if transcript levels were similar or higher than in hRA. The highest number of cardiac pacemaker-specific genes transcribed was observed in cells generated by protocol E (12/15 genes), even surpassing the number in cells differentiated by reference protocol A (11/15 genes). Cells generated by protocol D revealed the lowest number of active cardiac pacemaker-specific genes (8/15 genes).

In conclusion, cardiac pacemaker cells generated by protocol E displayed the best profile in terms of pacemaker specificity and hence were used exclusively for further characterization including immunocytochemistry and pharmacological testing. 

### 2.5. Immunocytochemical Analysis of Protocol E Derived Cardiac Pacemaker Cells

**Figure 5:**
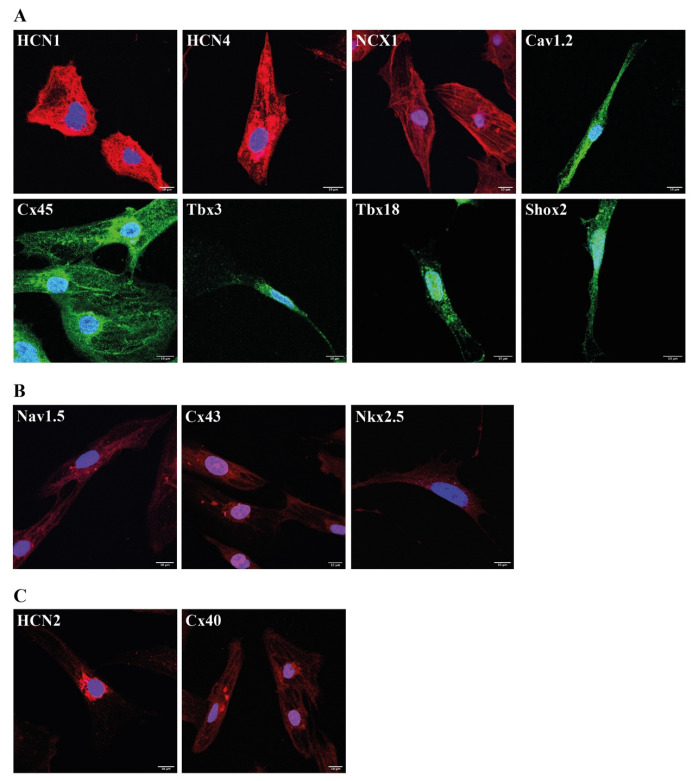
Immunocytochemical analysis of cardiac pacemaker cells differentiated by protocol E. Signals were visualized by confocal microscopy: (**A**): Pacemaker-specific markers. (**B**): Ventricular- specific markers. (**C**): Atrial-specific markers. Nuclei were counterstained with 4′,6-diamidino-2-phenylindole (DAPI). Scale bars = 10 μm.

Confocal imaging showed pronounced fluorescence signals of the cardiac pacemaker-specific markers HCN1, HCN4, NCX1, Ca_v_1.2, Cx45, Tbx3, Tbx18 and Shox2 (Figure 5A). By contrast, we observed only weak signals of the ventricular-like markers Na_v_1.5, Cx43 and Nkx2.5 (Figure 5B) and moderate signals of the atrial-like markers HCN2 and Cx40 (Figure 5C).

### 2.6. Spontaneous Beating Rate and Pharmacological Testing

**Figure 6:**
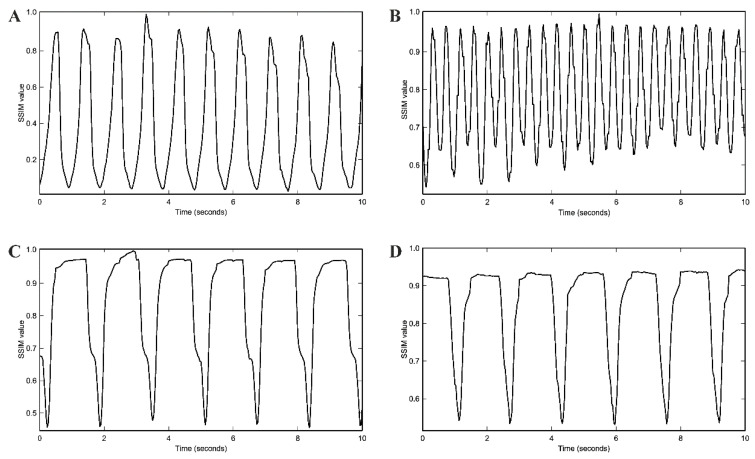
Spontaneous beating rate of cardiac pacemaker cells differentiated by protocol E. Cell video recording was performed using an inverted microscope equipped with a camera. Video analysis was realized by applying ImageJ software (Version 1.53) and structural similarity approach. (**A**): Basal spontaneous beating rate. (**B**): Spontaneous beating rate upon stimulation with 1 µM isoproterenol. (**C**): Spontaneous beating rate upon treatment with 1 µM carbachol. (**D**): Spontaneous beating rate upon treatment with 3 µM ivabradine. Abbreviation: SSIM: pair-wise similarity value.

The spontaneous beating rate of protocol E-derived cardiac pacemaker cells was assessed by video recordings and analyzed using a structural similarity approach (SSIM). Cells exhibited a spontaneous beating rate of 70.3 ± 4.8 beats per minute (bpm) (*n* = 30) (Figure 6A). Upon stimulation with 1 µM of the adrenergic substance isoproterenol, spontaneous beating rate significantly increased to 136.0 ± 7.3 bpm (*p* < 0.001) (*n* = 30) (Figure 6B). By contrast, treatment of cells with 1 µM of the cholinergic agent carbachol led to a significant decrease in spontaneous beating rate to 36.4 ± 2.8 bpm (*p* < 0.001) (*n* = 30) (Figure 6C). A similar effect was observed when we applied the HCN-channel inhibitor ivabradine in a concentration of 3 µM [48] that significantly decreased spontaneous beating rate to 40.4 ± 2.4 bpm (*p* < 0.001) (*n* = 30) (Figure 6D), pointing to the fact that there is no significant difference between carbachol and ivabradine regarding the ability to decrease beating rate (*p* = 0.357).

## 3. Discussion

Cardiac pacemaker devices are actually the only possibility to treat diseases affecting the hSAN. Due to their disadvantages including side effects and the missing neurohumoral regulation of rate response, the development of a biological pacemaker is of utmost importance [1,2,3]. Considering the increased oncogenic risk of a genetic therapeutic strategy [6,7], a cell-based approach appears to be very promising [4,5]. Owing to their pluripotency, hiPSC [8] represent a beneficial cell source for cell-based therapy [9]. Differentiation of hiPSC toward cardiomyocytes has already been successfully realized [10,11,12,13,14,15,16]. They consisted of the induction of mesoderm formation using either Wnt activation by CHIR99021, a small molecule inhibiting glycogen synthase kinase (GSK3) [15,16] or activation of the bone morphogenetic protein (BMP) and ACTIVIN A pathways [11,12,21]. After mesoderm formation, further differentiation into cardiomyocytes was achieved by inhibition of the Wnt pathways using the Wnt inhibitors IWP-2 or IWR-1 [15,16,21] or by culturing mesodermal cells after embryoid body formation in monocultures [11,12,14]. However, following these protocols, only 10% of the cells developed a pacemaker-like phenotype [17]. Overexpression of pacemaker-specific genes like *TBX3* [49] or *SHOX2* [50] after transfer into mouse pluripotent stem cells or of *TBX18* in cardiomyocytes of the working myocardium [6,7] resulted in a higher number of pacemaker-like cells, but, raised safety issues due to the increased risk of oncogenicity [21]. By contrast, in previous work, we were able to establish a protocol yielding a higher amount of cardiac pacemaker cells without the need for recurring genetic modifications [4]. 

Our protocol mainly consisted of two steps. In a first step, co-culture of hiPSC with END2 cells was performed to induce spontaneously beating cell clusters [10]. In a second step, beating cell clusters were transferred to an FBS-enriched cell culture medium to promote further differentiation toward a cardiac pacemaker-like phenotype [51]. Following our protocol, 63.4% of generated cells had a cardiac pacemaker-like phenotype [4]. 

However, the xenogeneic origin of END2 cells raises serious safety issues regarding future applications of cardiac pacemaker cells in a human clinical setting [18]. In addition, an END2 cell-mediated differentiation is less scalable than a differentiation by defined factors [19]. Therefore, we aimed to establish novel cell culture conditions that yield an increased differentiation efficiency comparable to our previous protocol [4] and substitute the use of END2 cells. 

We wanted to replace the first step of our previous protocol, namely the co-culture of hiPSC and END2 cells by xenogeneic-free small molecules involved in the activation and inhibition of the Wnt signaling pathway [15,16,17]. The second step, however, remained unchanged and consisted of the maturation of spontaneous beating cell clusters in FBS-enriched cell culture medium as previously described [4]. It has been recently shown that inhibition of NODAL by the small molecule SB431542 promotes the differentiation of hiPSC into cardiac pacemaker cells [20,21]. The addition of the NODAL inhibitor SB431542 to the Wnt inhibitor IWR-1 led to a 1.4–2.4-fold higher amount of hiPSC-derived cardiac pacemaker-specific cells compared to solely treatment with IWR-1 [20]. Furthermore, it was shown that administration of retinoic acid (RA), as well as a second application of the Wnt activator CHIR99021 after cell treatment by the Wnt inhibitor IWR-1, induced an atrial- and epicardial-like differentiation of hiPSC [21]. 

In consequence, our novel protocols B-D were based on activation and inhibition of Wnt signaling, supplemented either with RA and a second application of the Wnt activator CHIR99021 (protocol B) or with the NODAL inhibitor SB431542 (protocol C) or with all three substances (protocol D). Moreover, we wanted to examine the capability of the STEMdiff™ Atrial Cardiomyocyte Differentiation Kit from STEMCELL Technologies to differentiate hiPSC into cardiac pacemaker cells (protocol E), while our previously published END2 cell-based protocol for cardiac pacemaker-specific differentiation served as a reference (protocol A) [4]. 

To evaluate the differentiation efficiency of the protocols B-E, we compared the transcription profile of cardiac pacemaker cells generated by protocols B-E to that of cardiac pacemaker cells generated by reference protocol A [4]. Importantly, only spontaneously beating areas of 56-day-old cells were used for analysis. 

Although all protocols induced the upregulation of cardiac pacemaker-specific genes upon differentiation (Table 1), we observed significant differences regarding their differentiation efficiency. The administration of the NODAL inhibitor SB431542 (protocol C) was more efficient than a second application of the Wnt inhibitor CHIR99021 in combination with RA (protocol B) and led to an upregulation of 10 cardiac pacemaker-specific genes, whereas protocol B only upregulated 9 out of the 15 genes analyzed. Of note, protocol D displayed the worst performance with only eight genes upregulated, thus indicating that treatment with a combination of the Wnt inhibitor IWR-1 and NODAL inhibitor SB431542 followed by a combination of Wnt activator CHIR99021 and RA caused no enhanced differentiation efficiency toward cardiac pacemaker cells.

Despite being commercialized as a kit for atrial-like differentiation of hiPSC, protocol E led to significant overexpression of most of the cardiac pacemaker-specific genes analyzed, namely 12 out of 15. For comparison, reference protocol A [4] induced upregulation of 11 cardiac pacemaker-specific genes. Importantly, the pacemaker-specific transcription factor Shox2, which contributes to the development of cardiac pacemakers by upregulation of the pro-pacemaking transcription factors *Tbx3* and *Tbx18* [50,52], showed very high transcription levels in cardiac pacemaker cells generated by protocol E, which were clearly different from cells generated by the other protocols A, B, C and D. Similarly, transcription of *Tbx18*, which is critically involved in the formation of the sinus node head [53] showed highest levels in cells differentiated by protocols A and E, whereas *Tbx3*, important for the pacemaker-specific differentiation within cardiac tissue [53,54,55], showed highest levels in cells generated by protocol A, followed by cells differentiated by protocol E, thus clearly surpassing hRA levels. In contrast, much lower levels were observed in cells generated by protocols B-D.

Consistent with the transcription profiles, immunocytochemical analysis of cardiac pacemaker cells generated by protocol E displayed intense fluorescence signals of *Tbx3* and *Tbx18*, whereas expression of Nkx2.5, known to suppress hSAN formation [30] by downregulating Shox2 activity [56], was nearly undetectable. In conclusion, high *SHOX2* transcript levels, as well as intense Shox2 protein signals detected in cardiac pacemaker cells generated by protocol E, strongly indicate that this culture condition favors the differentiation of hiPSC toward a cardiac pacemaker cell lineage. 

It could be shown that Shox2 leads to an upregulation of the cardiac pacemaker-specific ion channel HCN4 [57], which next to HCN1, is responsible for the generation of the spontaneous depolarizing pacemaker current I_f_ in sinoatrial cells [25,32,33,36]. Similarly, *Tbx3* is known to activate the gene expression of *HCN4* and of the cardiac pacemaker-specific calcium channel *Cav3.1* [55]. Conversely, *Tbx3* suppresses the expression of myocardial genes like *Cx40*, *Cx43* and *SCN5A* [55]. Moreover, it could be shown that overexpression of *Tbx18* in hiPSC induced upregulation of *HCN4* and downregulation of *Kir2.1* [24]. The non-pacemaker-specific, ventricular-like transcription factor Nkx2.5, by contrast, suppresses *HCN4* as well as *TBX3* [58] and upregulates the atrial marker *Cx40* [59]. In line with this, protocol E led to pronounced transcription of the pacemaker-specific ion channels HCN1 and HCN4. These findings were confirmed by confocal analysis showing high protein levels of HCN1 and HCN4 in cells generated by protocol E. Similarly, we observed a strongly increased transcription for cardiac pacemaker-specific ion channels like NCX1 [39], Ca_v_1.2, Ca_v_1.3 and Ca_v_3.1 [40,41] in these cells, substantiated by intense immunocytochemical signals. In contrast to cells produced by reference protocol A and nearly lacking *HCN2* transcripts, protocol-E-induced cells showed much higher levels of the atrial marker *HCN2* [25]. Although *Cx40* [44,45] was higher transcribed in cells generated by protocol E than in cells differentiated by protocol A, its gene expression levels ranged, however, far below those of hRA or hSAN. Transcription of ventricular and non-pacemaker markers such as *cTnI*, *SCN5A* [42], *Cx43* [44,45] and K_ir_2.1 [60] remained relatively low too. Particularly, the very low transcription as well as the weak immunocytochemical signals of the ventricular-specific markers Na_v_1.5 [42] and Cx43 [44,45] underlined that culturing hiPSC following protocol E excludes a ventricular-like differentiation of hiPSC. Moreover, the appearance of only moderate fluorescence signals of the atrial markers HCN2 and Cx40 confirmed these observations. In contrast, the cardiac pacemaker-specific marker Cx45 was highly detected on mRNA as well as on protein levels. These findings clearly indicate that protocol E preferentially upregulates the expression of cardiac pacemaker-specific markers, even more efficiently than reference protocol A [4]. Moreover, the fact that the cardiac pacemaker-specific relationship between transcription factors and their target genes is maintained in cardiac pacemaker cells differentiated by protocol E, emphasizes once more the high potential of protocol E to differentiate hiPSC into cardiac pacemaker cells.

To assess the maturity of hiPSC-derived cardiac pacemaker cells and to exclude any proliferative activity during differentiation, we performed ATP-based cell proliferation assays. In contrast to hiPSC, which showed exponential growth, no significant cell proliferation or cell death could be recorded for cardiac pacemaker cells, independently of the used differentiation protocol. These findings are insofar important because they underline the stability of hiPSC-derived cardiac pacemaker cells, which is a prerequisite for a functioning biological pacemaker [61].

The rate responsiveness of sinoatrial cells to adrenergic and cholinergic substances is paramount for a proper cardiac pacemaker function [46,47]. Cardiac pacemaker cells generated by protocol E not only displayed high gene expression levels of adrenergic and cholinergic receptors but also revealed appropriate modulation by the adrenergic substance isoproterenol and the cholinergic substance carbachol. Stimulation with 1 µM isoproterenol led to a significant increase in spontaneous beating rate from 70.3 ± 4.8 bpm to 136.0 ± 7.3 bpm (*p* < 0.001) indicating an excellent adrenergic response. Conversely, treatment with 1 µM carbachol resulted in a significant decrease in spontaneous beating rate from 70.3 ± 4.8 bpm to 36.4 ± 2.8 bpm (*p* < 0.001), hence demonstrating an adequate response of cardiac pacemaker cells generated by protocol E to cholinergic agents. In addition, ivabradine has been shown to specifically inhibit I_f_ in cardiac pacemaker cells at a concentration of 3 µM [48]. Equally, in cardiac pacemaker cells generated by protocol E, 3 µM ivabradine lowered the spontaneous beating rate from 65.7 ± 1.3 bpm to 40.4 ± 2.4 bpm (*p* < 0.001). Of note, no significant changes in beating rate could be observed in hiPSC-derived cardiomyocytes, even if high concentrations of ivabradine up to 9 µM [62] were applied. 

## 4. Materials and Methods

### 4.1. Differentiation of hiPSC by Novel Cell Culture Protocols

hiPSC generated from human dermal fibroblasts (Catalog number: DF-F-ZB, BioCat GmbH, Heidelberg, Germany) [4] were used for the various differentiation assays. They were cultured in mTeSR™1 medium (STEMCELL Technologies Inc., Vancouver, BC, Canada) and grown on six-well plates (Greiner Bio-One GmbH, Frickenhausen, Germany), coated with Corning^®^ Matrigel^®^ hESC-Qualified Matrix (Corning, Inc., Corning, NY, USA) according to the manufacturer’s instructions. We developed four novel cell culture protocols (protocols B-E) and compared them to the END2 cell-based differentiation protocol (protocol A) [4] in terms of their ability to differentiate hiPSC into cardiac pacemaker cells. A schematic overview of the differentiation protocols A-E is depicted in Figure 1. To induce cardiac pacemaker-specific differentiation of hiPSC according to cell culture protocols B-D, hiPSC colonies of 90% confluency were mechanically dissociated and cultivated in RPMI 1640 medium (Thermo Fisher Scientific Inc., Waltham, MA, USA) containing B-27 supplement without insulin (Thermo Fisher Scientific Inc., Waltham, MA, USA) from days 0–6. On days 0 + 1, cell culture medium was supplemented with 6 µM of the Wnt activator CHIR 99021 (Sigma-Aldrich, St. Louis, MO, USA), and on days 3 + 4, the Wnt inhibitor IWR-1 was added at a concentration of 5 µM (Sigma-Aldrich, St. Louis, MO, USA). From day 7 to the start of spontaneous cell beating (days 10–12) cells were cultured in RPMI 1640 medium containing B-27 supplement (including insulin) (Thermo Fisher Scientific Inc., Waltham, MA, USA). From day 13 to day 56, spontaneously beating cells were cultured in FBS-enriched medium as previously described [4]. Cell culture protocols B-D differed from each other by following modifications: protocol B, 1 µM of retinoic acid (RA, Sigma-Aldrich) and 5 µM of CHIR 99021 were added on days 5 + 6; protocol C, 5 µM of the NODAL inhibitor SB431542 (Tocris Bioscience, Bristol, UK) were added on days 3 + 4; protocol D, 1 µM of RA and 5 µM of CHIR 99021 were added on days 5 + 6 in addition to 5 µM of SB431542 on days 3 + 4; protocol E used the STEMdiff™ Atrial Cardiomyocyte Differentiation Kit from STEMCELL Technologies (STEMCELL Technologies Inc., Vancouver, BC, Canada) to induce differentiation of hiPSC. For each novel protocol (B–E) differentiation assays were performed 16 times (*n* = 16 six-well plates).

### 4.2. Cell Proliferation Assays

To assess whether hiPSC continues to proliferate during differentiation, we performed adenosine triphosphate (ATP)-based cell proliferation assays using the BioFix^®^ Lumi ATP assay (Macherey-Nagel, Düren, Germany) according to the manufacturer’s instructions. Therefore, we analyzed cell proliferation of differentiated hiPSC (dhiPSC) cultured according to protocols B-E (*n* = 4 six-well plates for each protocol) on days 14, 28, 42 and 56 after the start of differentiation. For each ATP-based assay, we used dhiPSC from one well of a six-well plate, and each experiment was repeated six times.

### 4.3. RNA Isolation and cDNA Synthesis

On day 56, total RNA was isolated from dhiPSC cultured according to protocols B–E (*n* = 4 six-well plates for each protocol) by Trizol reagent (Thermo Fisher Scientific Inc.,) following the manufacturer’s instructions. Importantly, only spontaneously beating areas of dhiPSC, subsequently termed cardiac pacemaker cells, were used for RNA analysis. cDNA synthesis was realized by reverse transcription according to the manufacturer’s protocol (Superscript II; Thermo Fisher Scientific Inc.).

### 4.4. Quantitative Real-Time Polymerase Chain Reaction 

For quantitative real-time polymerase chain reaction (qRT-PCR), we used an ABS 7500 Realtime PCR System (Thermo Fisher Scientific Inc.) according to the manufacturer’s instructions. In detail, each well of a 96-well optical detection plate was loaded with 0.5 µL cDNA, 5 µL TaqMan Fast Universal Master Mix (Thermo Fisher Scientific Inc.), 0.55 µL 6-carboxyfluorescein (FAM)-labeled pre-designed TaqMan probes and primers (TaqMan Gene Expression Assays, Thermo Fisher Scientific Inc.) and 3.95 µL distilled RNAse-free water (Thermo Fisher Scientific Inc.). The used TaqMan probes and primers (Thermo Fisher Scientific Inc.) for the characterization of cardiac pacemaker cells are listed in Table 2. Transcript levels of analyzed genes were compared to a reference consisting of predesigned primers and probes for the housekeeping genes *glyceraldehyde 3-phosphate dehydrogenase* (*GAPDH*), *hypoxanthine-guanine phosphoribosyltransferase 1* (*HPRT1*) and *beta-actin* (*ACTB*) (all from Thermo Fisher Scientific Inc.). Assessment of gene expression relative to the combined reference of *GAPDH*, *HPRT1* and *ACTB* was calculated using a modified threshold cycle (CT) quantification method as described elsewhere [63]. All PCR reactions were carried out in triplicates, and the results were calculated as the average of the triplicates. The relative abundance of gene transcripts in cardiac pacemaker cells generated by protocols B–E was compared to the already published relative abundance of gene expression in cardiac pacemaker cells differentiated by protocol A [4]. Finally, the relative abundance of gene expressions in cardiac pacemaker cells generated by protocols A–E was compared to the relative abundance of gene transcripts in the human right atrium (hRA) or human sinoatrial node (hSAN), which has already been published [4]. hSAN transcript levels were determined [4] by multiplying hRA transcript levels with a previously published hRA and hSAN gene expression ratio [25].

### 4.5. Immunocytochemistry of hiPSC-Derived Cardiac Pacemaker Cells

dhiPSC differentiated by protocol E were used on day 56 of cell culture for immunocytochemical analysis. For dissociation into single cells, 56-day-old spontaneous beating areas of dhiPSC cultured on six-well plates (*n* = 4) in Maintenance medium (STEMCELL Technologies) were treated with STEMdiff™ Cardiomyocyte Dissociation Kit (STEMCELL Technologies Inc.) according to the manufacturer’s instructions. Dissociated cardiac pacemaker cells were seeded on Matrigel-coated (Corning^®^ Matrigel^®^ hESC-Qualified Matrix) 0.5 cm diameter coverslips (Menzel-Gläser, Gerhard Menzel GmbH, Braunschweig, Germany) in 24-well plates (Greiner Bio-One) (*n* = 4) and cultured for one day in STEMdiff™ Cardiomyocyte Support Medium (component of STEMdiff™ Cardiomyocyte Dissociation Kit). After 24 h, cell culture medium was switched to Maintenance medium. For immunocytochemistry, dissociated cardiac pacemaker cells were treated with 0.5% TritonTM X-100 (Sigma-Aldrich)/PBS solution for 15 min, followed by 0.1 M glycine (Merck)/PBS solution for 60 min before being incubated in blocking solution (PBS with 2% bovine serum albumin; Sigma-Aldrich) for 2–3 h. Afterward, cells were incubated with primary antibodies, diluted 1:200 in blocking solution, overnight at 4 °C. Subsequent primary antibodies were used: anti-HCN1 (IgG, rabbit, Alomone Labs, Ltd., Jerusalem, Israel), anti-HCN2 (IgG, rabbit, Alomone Labs, Ltd.), anti-HCN4 (IgG, rabbit, Alomone Labs, Ltd.), anti-NCX1 (IgG, rabbit, Alomone Labs, Ltd.), anti-Ca_v_1.2 (IgG, mouse, Abcam, Cambridge, UK), anti-Na_v_1.5 (IgG, rabbit, Alomone Labs, Ltd.), anti-Cx40 (IgG, rabbit, Alomone Labs, Ltd.), anti-Cx43 (IgG, rabbit, Alomone Labs, Ltd.), anti-Cx45 (IgG, mouse, Abcam) and anti-*Tbx3* (IgG, mouse, Abcam), anti-*Tbx18* (IgG, mouse, Santa Cruz Biotechnology Inc., Dallas, TX, USA), anti-Shox2 (IgG, mouse, Abcam) and anti-Nkx2.5 (IgG, rabbit, Abcam). The following day, cardiac pacemaker cells were washed three times with PBS and incubated with secondary antibodies, diluted 1:200 in blocking solution, at 4 °C for 4 h. Secondary antibodies included MFP555 goat anti-rabbit IgG (H + L) (MoBiTec GmbH, Göttingen, Germany) and MFP488 goat anti-mouse IgG (H + L) (MoBiTec GmbH). After three washes with PBS, nuclei were counterstained with 1 μg/mL 4′,6-diamidino-2-phenylindole (DAPI, Sigma-Aldrich, St. Louis, MO, USA) for 30 min. Afterward, cells were washed three times with PBS and mounted with Citifluor glycerol/PBS-solution AF1 (Agar Scientific Ltd., Stansted, UK). They were visualized by confocal microscopy using the Olympus confocal microscope FV3000 (Olympus, Tokyo, Japan). Editing and analysis of confocal images were performed with ImageJ software (Version 1.53) (National Institutes of Health, Bethesda, MD, USA).

### 4.6. Assessment of Spontaneous Beating Rate of Cardiac Pacemaker Cells by Movement Frequency Analysis with Structural Similarity Approach

To assess the spontaneous beating rate of cardiac pacemaker cells differentiated by protocol E (*n* = 24 wells of six-well plates), cell video recording was performed using the Leica DMi1 inverted microscope equipped with the camera Leica MC120 HD (Leica Camera AG, Wetzlar, Germany). Video analysis was realized by applying ImageJ software (Version 1.53). To measure and analyze the movement (beating) frequency of spontaneous beating cardiac pacemaker cells from the movie, we used a structural similarity approach implemented as a custom Fiji (Laboratory for Optical and Computational Instrumentation, University of Wisconsin-Madison, Madison, WI, USA) Groovy script. The structural similarity measurement was inspired by Wang et al. [64] and slightly modified to better fit the brightness and contrast and signal-to-noise ratio of our data. More specifically, we took a given time frame, or the time average of the whole movie as our reference frame. Then we computed the pair-wise similarity (SSIM) value, between the reference frame and each time frame of the whole movie. The pair-wise similarity score can be computed either using the whole field of view of the movie or a selected area as region-of-interest (ROI). 

### 4.7. Pharmacological Testing

To analyze the influence of pharmacological substances on the spontaneous beating rate, we treated cardiac pacemaker cells differentiated by protocol E with 1 µM isoproterenol hydrochloride (Sigma-Aldrich) (*n* = 6 wells of a six-well plate), 1 µM carbachol (Sigma-Aldrich) (*n* = 6 wells of a six-well plate) and 3 µM ivabradine hydrochloride (Sigma-Aldrich) (*n* = 6 wells of a six-well plate).

### 4.8. Statistical Analysis

For each protocol (B-E), hiPSC differentiation was performed on 16 six-well plates. Four six-well plates were used for RNA extraction and subsequent qRT-PCR analysis and further four six-well plates for the cell proliferation assays. For protocol E, four six-well plates of dhiPSC were used for immunocytochemistry and further four six-well plates for pharmacological testing and video analysis. For each well, five video recordings were performed.

GraphPad Prism (Version 9.2.0, GraphPad Prism Software, Inc., San Diego, CA, USA) was used for statistical analysis. Data of multiple groups were compared by one-way ANOVA, followed by a Tukey post hoc test. Moreover, a two-tailed unpaired Student’s *t*-test was performed for pairwise comparison. Differences were judged significant at a level *p* < 0.05. Arithmetic mean as well as the standard error of the mean (SEM) were used for data presentation.

## 5. Conclusions

The best cardiac pacemaker-specific transcription profile was attained in cells generated by protocol E providing high levels of gene expression in 12 of 15 cardiac pacemaker-specific genes, while reference protocol A only induced upregulation of 11 cardiac pacemaker-specific genes [4]. Moreover, except for *HCN2* and *Cx40*, we observed no relevant transcriptional induction of genes representative of atrial- or ventricular-like markers in cells generated by protocol E.

Although developed for atrial-like differentiation of hiPSC, protocol E led to a significant induction of a high number of genes essentially required for the differentiation of hiPSCs to cardiac pacemaker cells (*HCN4*, *HCN1*, *Ca_v_1.2*, *Ca_v_1.3*, *Ca_v_3.1*, *TBX3*, *TBX18* and *SHOX2*) with the absolute advantage to obviate the need for END2 cells.

## Figures and Tables

**Table 1:** Assessment of pacemaker-specific gene expression in cardiac pacemaker cells.

	Diff. Prot. A	Diff. Prot. B	Diff. Prot. C	Diff. Prot. D	Diff. Prot. E
***TBX3***	+	+	+	−	+
***TBX18***	+	−	−	−	+
***SHOX2***	−	−	−	−	+
***BMP4***	+	−	+	+	−
***HCN1***	+	−	−	−	+
***HCN4***	+	+	+	+	+
***NCX1***	+	+	+	+	+
***Ca_v_1.2***	+	+	+	−	+
***Ca_v_1.3***	+	+	+	+	+
***Ca_v_3.1***	+	+	+	+	+
***Cx45***	−	−	−	−	−
***α1a-AR***	−	+	+	+	+
***β1-AR***	−	−	−	−	−
***β2-AR***	+	+	+	+	+
***CHRM2***	+	+	+	+	+
Total	11/15	9/15	10/15	8/15	12/15

**Table 2:** Overview of the used TaqMan probes and primers.

Analyzed Gene		Primer Accession Number (TaqMan Probe and Primer)
Abbreviation	Written-Out Form	
*GAPDH*	glyceraldehyde-3-phosphate dehydrogenase	Hs99999905_m1
*HPRT1*	hypoxanthine phosphoribosyltransferase 1	Hs01003270_m1
*ACTB*	actin beta	Hs010606665_m1
*TBX3*	T-box transcription factor 3	Hs00195612_m1
*TBX18*	T-box transcription factor 18	Hs01385458_m1
*SHOX2*	short stature homeobox 2	Hs00243203_m1
*BMP4*	bone morphogenetic protein 4	Hs00370078_m1
*TBX5*	T-box transcription factor 5	Hs01052563_m1
*MEF2C*	myocyte enhancer factor 2C	Hs01554602_g1
*NKX2.5*	NK2 homeobox 5	Hs00231763_m1
*cTnI*	troponin I3, cardiac type	Hs01036382_g1
*HCN1*	hyperpolarization activated cyclic nucleotide gated potassium channel 1	Hs00395037_m1
*HCN2*	hyperpolarization activated cyclic nucleotide gated potassium channel 2	Hs00606903_m1
*HCN4*	hyperpolarization activated cyclic nucleotide gated potassium channel 4	Hs00175760_m1
*NCX1*	sodium/calcium exchanger protein	Hs01062258_m1
*Ca_v_1.2*	calcium voltage-gated channel subunit alpha1 C	Hs00167681_m1
*Ca_v_1.3*	calcium voltage-gated channel subunit alpha1 D	Hs00167753_m1
*Ca_v_3.1*	calcium voltage-gated channel subunit alpha1 G	Hs00367969_m1
*SCN5A*	sodium voltage-gated channel alpha subunit 5	Hs00165693_m1
*K_ir_2.1*	potassium inwardly rectifying channel subfamily J member 2	Hs00542478_m1
*hERG*	potassium voltage-gated channel subfamily H member 2	Hs00265315_m1
*K_v_4.3*	potassium voltage-gated channel subfamily D member 3	Hs00542597_m1
*Cx45*	gap junction protein gamma 1	Hs01087407_s1
*Cx40*	gap junction protein alpha 5	Hs99999170_s1
*Cx43*	gap junction protein alpha 1	Hs00748445_s1
*α1a-AR*	adrenoceptor alpha 1A	Hs00169124_m1
*β1-AR*	adrenoceptor beta 1	Hs02330048_s1
*β2-AR*	adrenoceptor beta 2	Hs00240532_s1
*CHRM2*	cholinergic receptor muscarinic 2	Hs00265208_s1

## Data Availability

The data presented in this study are available on request from the corresponding author.
